# Insight into the Sustainability of the Mediterranean Diet: The Water Footprint of the Recommended Italian Diet

**DOI:** 10.3390/nu15092204

**Published:** 2023-05-05

**Authors:** Alessandra Bordoni

**Affiliations:** 1 Department of Agricultural and Food Sciences (DISTAL), University of Bologna, Piazza Goidanich 60, 47521 Cesena, FC, Italy; alessandra.bordoni@unibo.it; Tel.: +39-0547338955; 2 Interdepartmental Centre for Industrial Agri-Food Research (CIRI), University of Bologna, Via Quinto Bucci 336, 47521 Cesena, FC, Italy

**Keywords:** water footprint, Mediterranean Diet, Italian Dietary Guidelines, sustainability

## Abstract

At present, as we face climate change and natural resource scarcity, one of the major challenges linking humans and the environment is to ensure sufficient, nutritious, safe, and affordable food for a rapidly growing world population. In a nutshell, “feed the world without destroying it”. The water footprint (WF), i.e., the withdrawals of fresh water necessary to produce one kilogram of food product, is one of the key indicators of the environmental impact of diets. In this work, the WF of the food patterns suggested by the Italian Food Based Dietary Guidelines, considered a model of the Mediterranean Diet, was evaluated for the first time. The data reported here clearly demonstrate that the suggested Italian dietary patterns have a low WF, the reduction of which by replacing animal foods with plant foods is limited because the suggested consumption of meat is already low. Consumer choice in the consumption of specific products within a food group could further reduce the WF of the diet, underlining the need to provide correct information not only to consumers but also to farmers and producers to encourage them to make water-saving choices.

## 1. Introduction

Sustainable diets are defined as diets with a low environmental impact and those that contribute to food and nutrition security and to a healthy life for present and future generations [1]. Accordingly, the goal of food systems in the 21st century should be to provide food security for all while minimizing the environmental impact of food production. Therefore, a “correct” diet should integrate the different dimensions of sustainability, i.e., health/nutrition, economics, society, and the environment. This integration is not as straightforward as is sometimes suggested, and some crucial aspects of this complex issue should be carefully considered. One of these aspects is related to metrics and measure, since the contributions of the food system to climate change are usually measured in greenhouse gas emissions (GHGEs). In reality, the analysis of the environmental impacts of diets must expand beyond GHGEs [2], including the impact on energy and fuel use, land capacity, water, and biodiversity as well as the impact of producing, transporting, and wasting food on each of these components [3]. To this aim, a number of additional key indicators of environmental sustainability in food systems have been identified [4]. These include the water footprint (WF), i.e., the withdrawals of fresh water necessary to produce one kilogram of food product. Since approximately 70% of freshwater globally is used for agricultural production (food and non-food), it is clear that food security also depends on the availability of freshwater resources and that understanding the impact of food production and population-level dietary patterns on water use is critical.

When it is necessary to integrate the health/nutrition dimension and the environmental dimension of diet sustainability, the common practice of calculating the environmental footprint, including the WF, of food production per kilogram of food product has limitations as there is no human requirement for a daily “weight” of food. Therefore, a calculation of the environmental cost in relation to energy needs and nutrient requirements is mandatory for a better understanding of the sustainability of a diet. Due to the multiplicity of different diets, the starting point for calculating sustainability should be its assessment using reference diets that provide the amount of energy and nutrients needed for a healthy life.

The population-level assessment of energy and nutrient requirements has enabled the development of nutrient-based recommendations, called dietary reference intakes (DRIs), which are country-specific in most high-income countries [5,6]. The DRIs were translated into the Food-based Dietary Guidelines (FBDG), which indicate food consumption patterns that meet the energy and nutrient requirements established by the DRIs. National FBDGs considered cultural habits, food preferences, and portion sizes, thus integrating the social dimension of diet sustainability.

The fourth edition of the “Italian FBDGs (IDGs) for Healthy Eating” was published in 2019 and considered the whole dietary pattern. The entity of the portions of the different foods was defined, as well as their frequency of consumption, to attain a complete and balanced diet with commonly used foods that are easily available and adhere to Italian culture and tradition [7]. The IDGs were explicitly based on the Mediterranean diet (MedD) and also included sustainability recommendations on food selection, discouraging foods that are considered to have a negative impact on health and the environment and giving priority attention to food loss and waste [8].

The inclusion of sustainability in the FBDGs is in progress in several countries [9]. However, the degree of integration of the health and environmental dimensions of sustainability in the dietary patterns suggested by the IDGs, considered as models of the MedD, has never been evaluated.

In this study, the WF of the dietary patterns suggested in the IDGs for the adult Italian population was calculated to verify whether their adoption complies with a low-water-consumption goal. To clarify, it is not trivial, as in the past, an increase in nutritional quality was not associated with a decrease in GHGEs [10], and a higher consumption of water—and to a lesser extent, soil—has been associated with “sustainable diets” by Jarmul S et al. [11].

While confirming the sustainability of the Italian MeD model in relation to water consumption, the results reported here highlight that some compromises, filings, or refinements may be necessary. It is therefore necessary to sensitize the agri-food chain and consumers, encouraging them to make water-saving choices.

## 2. Materials and Methods

The dietary patterns suggested by the IDGs were considered. In the IDGs, the serving sizes and the suggested number of daily/weekly servings that should be consumed by Italian healthy adults are reported for the different food categories, considering three different energy intakes: low (1500 kcal/day); medium (2000 kcal/day); high (2500 kcal/day).

In each food category, the weight (in g) of the serving was multiplied by the number of recommended servings to obtain the weight of the food to be consumed per week. The WFs of the various food products were calculated by averaging the data reported by Mekonnen and Hoekstra [12,13] in each food category, taking into account Italian eating habits and the available data. In the IDG, the quantity of food that corresponds to a serving is expressed as the weight of the edible part, while the WF is calculated as the production weight; therefore, a conversion was made where necessary (e.g., fruits), based on data reported in [14]. In details:

Food group 1 (meat, terrestrial and aquatic animals, and eggs). The WF of red meat was considered the mean WF of beef and pork, while the WF of poultry was considered for white meat since other types of white meat are consumed less in Italy. Since the paper by Mekonnen and Hoekstra [13] did not include information on the WF for fish and seafood, data from Bostock et al. [15] were used. The WFs for the types of fish and seafood most frequently consumed in Italy (salmon, sea bass, sea bream, halibut, sole, cod, tuna, and mussels) were averaged, considering the water consumption per unit of production in aquaculture systems. As aquaculture was reported to have contributed 43% of the human consumption of aquatic animals in 2007 and was expected to grow further to meet future demand [15], 50% of the suggested consumption was considered to come from aquaculture. The WF of wild fish was considered to be 0 L/kg [16].

Food group 2 (milk and dairy products). The IDGs suggest the consumption of 3 servings/week of cheese. Since the serving size is different for soft cheese (100 g) and hard cheese (50 g), and soft cheese such as mozzarella is frequently consumed in Italy, the consumption of 2 servings of soft cheese and 1 serving of hard cheese was considered for a total of 250 g of cheese per week.

Food group 3 (cereals and tubers). As the WFs for bread analogues (breadsticks, crackers, etc.) were not available, the suggested weekly consumption of these products (30 g) was added to the bread consumption. The WF of sweet baked goods was calculated on the basis of the WFs of the corresponding ingredients in two traditional recipes: croissants and biscuits. Oat flakes were considered for the WF of breakfast cereals.

Food group 4 (pulses). The value used for the calculation was the average value of the WFs of dry beans, peas, chickpeas, and lentils, which represent the most frequently consumed pulses in Italy.

Food group 5 (fats). The IDGs recommend consuming 2–4 servings/day of fats, depending on energy intake, advising consumers to prefer vegetable oils, mainly extra virgin olive oil (EVO). Thus, 1.5–3 servings and 0.5–1 serving were considered for vegetable oils and butter, respectively. Regarding vegetable oils, the WFs of those most consumed in Italy (EVO, olive oil, corn oil, and sunflower oil) were averaged and used for the calculation.

Food groups 6 and 7 (fruits and vegetables). The WFs of 15 types of fruit (peach, orange, tangerine, banana, apple, pear, grapefruit, apricot, plum, cherry, strawberry, table grape, watermelon, kiwi, and pineapple) were averaged and used for the calculation. These fruits were chosen not only because they are widely consumed but also because many of them are available in Italy almost all year round. As there is a specific recommendation for the consumption of nuts in the IDGs, the average WFs of shelled peanuts, walnuts, pistachios, almonds, and hazelnuts were included in the calculation. As far as vegetables are concerned, the IDGs suggest the consumption of 2.5–3 portions per day (17.5–21 portions/week), depending on energy intake. Since the serving size is different for leafy vegetables (80 g) and other vegetables (200 g), a weekly consumption of 7 servings of leafy vegetables and 10.5–14 servings of other vegetables were assumed based on the Italian dietary habits. The WF of leafy vegetables considered in the calculation was the mean of the WFs of lettuce, spinach, and cabbage, while the WF of other vegetables was the mean of the WFs of tomatoes, cauliflower, broccoli, artichokes, asparagus, eggplants (aubergines), peppers, green beans, and carrots.

“Indulgence” foods i.e., rich-tasting foods that need to be limited in the diet. The IDGs provide indications only for sugar, honey, and jams, and simply indicate “consume sparingly” for other foods. Consequently, initially only the recommendations for sugar and jam consumption were taken into account and the corresponding WF included in the calculation. The WF for sugar was the average of the WFs for beet and brown sugar, and the WF of jam was calculated based on a 35% fruit and 40% sugar content. Subsequently, the consumption of 1 portion/day of wine or beer was also considered, and the mean WF was calculated considering the different serving size of the two alcoholic beverages (125 and 330 mL, respectively). Furthermore, the consumption of coffee and tea was fixed at 1 cup per day, and the mean WF was calculated and included considering the different serving size of the two drinks (50 and 150 mL, respectively).

This study had a correlation research design, aiming at identifying relationships between variables without implying causation.

## 3. Results

The WFs of the dietary patterns recommended by the IDGs are reported in Table 1, Table 2 and Table 3.

The daily WFs of the three suggested diets were 2192, 2828, and 3250 L/per capita. Although in the range of dietary patterns in Europe (2873–3792 L/d per capita) [17], the WFs of the medium and high energy diets were higher than the WF estimated by Vanham D et al. [18] for the MedD in Italy (based on an intake of 2000 kcal for women and 2500 kcal for men), corresponding to 2571 L/day per capita. We assume that this difference is attributable to a different calculation since in the present study, the recommended serving size, expressed as the weight of the edible part of the food, was converted to the weight of the food at production. As shown in Table 1, Table 2 and Table 3, for some foods, this conversion led to an increase in the amount of food from which the WF was calculated. For example, the weight of one serving (as edible part) of potatoes (200 g) corresponds to 241 g at production, and the WF was calculated on the production weight.

**Figure 1:**
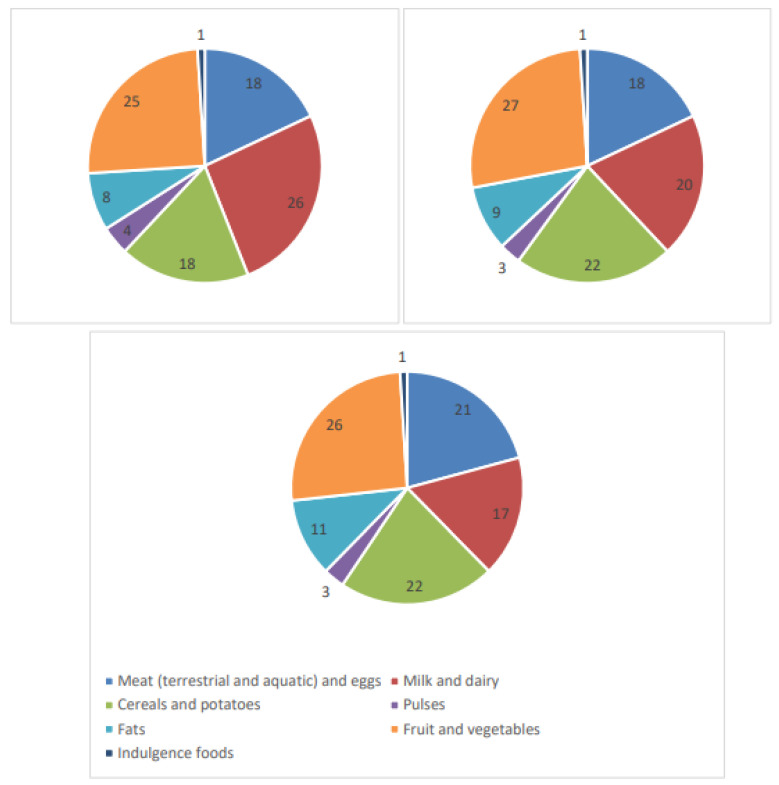
Contribution of the different food groups to the WF of the suggested Italian MedD. A = low-energy (1500 kcal/d) diet; B: medium-energy (2000 kcal/d) diet; C: high-energy (2500 kcal/d) diet.

The contribution of each food group to the total WF was then calculated, and it is shown in Figure 1.

Fruit and vegetables were one of the main contributors to the WF of the diets (25–27% of total WF). Regarding fresh fruit, the average WF of the various types of fruit frequently consumed in Italy was initially considered in the calculation. Since there is considerable variability between the types of fruit (from 255 L/kg for pineapples to 2180 L/kg for plums), the total WF was then recalculated, considering only fruits with a WF lower than 1000 L/kg (average value 601 L/kg). Likewise, vegetables with a WF higher than 600 L/kg were excluded, and the new mean value (287 L/kg) was used for the recalculation. A 6% decrease in the WF was thus achieved, indicating that shifting consumer choices towards low-WF fruit and vegetables could lead to a significant increase in sustainability with no impact on the nutritional value of the diet. In confirmation of this, it is worth noting that a different proportion in the consumption of leafy and non-leafy vegetables, ranging from “all leafy” to “no leafy”, would modify the daily WF of the 1500 kcal diet from −6% (all leafy) to +4% (no leafy), of the 2000 kcal/diet from −4% (all leafy) to +3% (no leafy), and of 2500 kcal/diet from −5% (all leafy) to +3% (no leafy). The suggested daily intake of nuts, which are rich in nutrients and an important source of protein, was included in the calculation from the outset. Since the EAT-Lancet Universal Healthy Reference Diet [19] recommends an increase in the consumption of healthy foods, including nuts, which are water-intensive products [12], the increase in the WF of the diets was recalculated considering the additional intake of 1 serving (30 g)/shelled nuts per day. Daily nut consumption increased the WFs of the dietary patterns by 12%, 8%, and 6% for the low-, medium-, and high-energy diets, respectively. Again, consumer choice could make a difference as the WF of peanuts is about 1/3 of the WF of tree nuts.

Group 3 foods were the second largest contributor to the total WF in the medium- and high-energy diets. The high frequency of consumption and the serving size of pasta is one of the typical characteristics of the Italian MedD in which pasta, in combination with bread, provides most of the amount of starch necessary to cover 45–60% of the energy requirement [6]. The WFs of pasta, rice, and barley were considered and averaged for the calculation. Although the WF of pasta is lower than the WFs of rice and barley, the differences are minimal, and the consumption of one instead of the other would have little impact on the WF of the diet.

Since the suggested number of servings of group 2 foods is the same regardless of energy intake to cover calcium needs, milk and dairy products were the first contributor to total WF in the 1500 kcal/d diet. As reported in the Methods Section, the WF of the group 2 foods was calculated considering 2 servings/week of soft cheese and 1 serving/week of hard cheese. A different proportion of soft and hard cheese consumption (from 3 servings/week of soft cheese to 3 servings/week of hard cheese) would have a limited impact on the total WF of the diet, i.e., 1–3% of the reported value. Consumers have an increasing tendency to consume foods derived from plants, including imitations of milk. Consumer motivations and the nutritional and health implications of consuming plant-based beverages instead of milk have been reported elsewhere [20,21] and are outside the scope of this study. However, it is worth noting that the replacement of cow milk with soy milk would significantly increase the daily WF of the diets to 3221, 3857, and 4279 L per capita (+47%, 36% and 32%, respectively), indicating that the replacement of foods of animal origin with foods of plant origin does not always lead to a more sustainable diet.

Group 1 foods contributed only about 1/5 (18–21%) to the WF of the diets. As the consumption of meat (red + white) is recommended 2–3 times a week by the IDGs, this confirms suggestion of the growing body of literature that low-meat diets have a low environmental impact and low resource requirements [22,23,24]. Again, consumer choice could make a difference, as the WF of beef is approximately three times higher than that of pork or poultry. To verify whether the transition to a diet richer in plant foods could improve environmental sustainability, the suggested consumption of meat (red and white) was replaced by pulses in such quantities as to guarantee the same protein intake. This change in dietary patterns resulted in a 6–7% decrease in the WF of the diets. By replacing the consumption of meat from both terrestrial and aquatic animals with the consumption of pulses, while maintaining a similar quantity of proteins in the diet, the reduction in the WFs of the dietary patterns was less consistent and amounted to between 2 and 3%.

Group 5 foods contributed about 10% of the total WF of the suggested diets. For the calculation, the mean WF value of olive oil and the most-consumed seed oils in Italy was used. Although the WF of olive oil, a pillar of the Mediterranean diet, is higher than that of seed oils, it excluding it from the calculation was not considered due to its high nutritional value.

**Figure 2:**
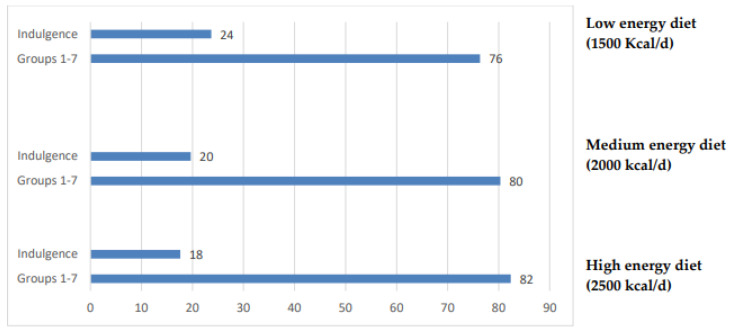
Percent contribution of indulgence foods to the WF of the suggested Italian MeD.

Based on the IDGs, indulgent foods contributed a very small percentage of the total WF of the diets. Since the guidelines do not recommend but indicate the permitted frequency of consumption of beer/wine and tea/coffee, the contribution of these beverages was then included in the indulgence food group and the corresponding contribution to the dietary WF was calculated. As shown in Figure 2, the consumption of indulgence foods, although in permitted amounts (one serving of wine or beer and three servings of coffee or tea), significantly increased the WFs of the diets.

## 4. Discussion

The existence of the “food gap”, i.e., the increase in the amount of food that the world will require in 2050, is well recognized, and one of the main challenges linking humans and the environment is ensuring sufficient, nutritious, safe, and affordable food for all. According to the Food and Agriculture Organization of the United Nations (FAO), we need sustainable diets with a low environmental impact. In a water–energy–food–ecosystem nexus, a shift to diets that are both nutritious and sustainable is needed to achieve the United Nations Sustainable Development Goals (SDGs) 2 on food security and 6 on water security [25]. The food and water sectors are intrinsically linked [26,27], and this link is crucial for the MedD. Indeed, the Mediterranean diet is a territorial diet that has its roots entrenched in the history of the Mediterranean Sea and its region [28], which already face moderate-to-high water stress, especially during summer months [29].

In a recent systematic review [30], the MedD showed a lower carbon footprint and WF than Western diets. The data reported here confirm this, showing that the MedD patterns suggested by the IDGs have low WFs. It should be noted that the diets suggested by the IDGs do not include indulgent foods such as alcoholic beverages or nervine drinks, the consumption of which is considered admissible in limited quantities. The inclusion of these limited amounts in the calculation significantly increased the WFs of the diets, and this should be an additional reason for their limitation. For completeness of information, it should be noted that the WF of a portion of coffee is about 1/5 of the WF of a portion of tea. Furthermore, the WF of one serving of wine is approximately 2.5 times lower than that of one serving of beer.

This study also highlighted that in dietary patterns in which the consumption of foods of animal origin is already low, their replacement with plant foods has a limited impact on the WF which is mainly related to the replacement of meat. Although a reduction in food of animal origin is usually reported as having less impact on the environment and on resource needs [17,24], it may not correspond to a lower water consumption, especially if animal foods are replaced with plant foods that are more dependent on irrigation [31]. This was clearly shown when cow’s milk was replaced by soy milk in the WF calculation. Furthermore, it was highlighted that consumer choice in the consumption of specific products within a food group could further reduce the WF of the diet, underlining the need to provide correct information to consumers. In fact, despite several studies aimed at understanding consumers’ perception of food sustainability [32,33,34], there is still a lack of information on how to increase their correct perception.

This study has limitations. First, the data used to calculate the WFs of the diets [12,13] represent average values for the period 1996–2005. To our knowledge, this is the highest quality data available on food WFs, but the growing interest and attention of the agri-food industry in water use may have resulted in less water being wasted. For example, the increasing use of the recirculating aquaculture system (RAS) may have significantly reduced the WF of aquaculture products [35]. Secondly, water consumption related to food processing and logistics was not considered in the calculation as a complete data set was not available. Therefore, the WF of dietary patterns was underestimated, also due to food waste and losses. For some foods, the difference in weight between the product at production and the edible part is significant, and by normalizing the two values for the calculation, it was possible to include the “almost mandatory” losses (for example, potato peel) in the total WF. Other food, industrial, and domestic losses could not be considered.

Beyond these limitations, this is the first report that calculates the WFs of dietary patterns suggested at the population level, highlighting their sustainability in relation to water consumption and confirming that a diet that includes a limited intake of foods of animal origin it is desirable for both health and the environment.

Since the MedD includes a high consumption of plant foods, it becomes strategic to think at 360 degrees on the water supply chain in order to optimize the WF of the agricultural sector, promoting the adoption of more efficient irrigation techniques and encouraging the reuse of wastewater, as also indicated by the recent European legislation on the reuse of wastewater (EU Regulation 741/2020). It is hoped that increasing the knowledge about the WF of foods and diets will raise awareness in the agri-food chain and among consumers, prompting them to opt for water-saving production and to adapt their dietary habits to the suggested healthy patterns, benefiting their health both directly and indirectly.

## Figures and Tables

**Table 1:** Water footprint of the low-energy (1500 kcal/day) dietary pattern.

Food Category	Serving Size (g of Edible Portion)	Suggested Consumption (Serving/Week)	Suggested Consumption (g/Week) ^a^	WF (L/kg or L/L)	WF of the Suggested Consumption (L/Week)
**Food group 1—meat (terrestrial and aquatic animals) and eggs**					
Red meat	100	1	113	10,701	1209
White meat	100	1	143	4325	618
Fish/seafood	150	2	428	2543	544
Processed fish	50	0	0	2543	0.0
Eggs	50	2	115	3265	375
**Food group 2—milk and dairy**					
Milk/yogurt	125	21	2625	1020	2678
Soft cheese	100	2	200	5060	1012
Hard cheese	50	1	50	5060	253
**Food group 3—cereals and potatoes**					
Bread	50	17.5	875	1608	1407
Bread analogue	30	1	30	1608	48
Pasta, rice, barley	80	10.5	840	2108	1180
Sweet bakery (croissant)	50	0.25	12.5	4062	51
Sweet bakery (biscuits)	30	0.25	7.5	3057	23
Breakfast cereals	30	0.5	15	2416	36
Potatoes	200	1	241	287	69
**Food group 4—pulses**					
Dry pulses	50	3	150	4271	641
**Food group 5—fats**					
Vegetable oil	10	10.5	105	9631	1011
Butter	10	3.5	35	5553	194
**Food groups 6 and 7—fruit and vegetables**					
Fresh fruit	150	14	2716	819	2225
Nuts, shelled	30	1	30	10,245	307
Leafy vegetables	80	7	742	255	189
Other vegetables	200	10.5	2919	402	1173
**Indulgence food**					
Sugar	5	7	35	1351	47
Jam	20	1	20	2675	54
**Total**					**15,347**

^a^ Converted considering the percentage of the edible part, when needed.

**Table 2:** Water footprint of the medium-energy (2000 kcal/day) dietary pattern.

Food Category	Serving Size (g of Edible Portion)	Suggested Consumption (Serving/Week)	Suggested Consumption (g/Week) ^a^	WFP (L/kg or L/L)	WFP of the Suggested Consumption (L/Week)
**Food group 1—meat (terrestrial and aquatic animals) and eggs**					
Red meat	100	1	113	10,701	1209
White meat	100	2	286	4325	1237
Fish/seafood	150	2	428	2543	544
Processed fish	50	1	50	2543	64
Eggs	50	3	172.5	3265	563
**Food group 2—milk and dairy**					
Milk/yogurt	125	21	2625	1020	2678
Soft cheese	100	2	200	5060	1012
Hard cheese	50	1	50	5060	253
**Food group 3—cereals and potatoes**					
Bread	50	24.5	1225	1608	1970
Bread analogue	30	1	30	1608	48
Pasta, rice, barley	80	10.5	840	2108	1770
Sweet bakery (croissant)	50	1	50	4062	203
Sweet bakery (biscuits)	30	1	30	3057	92
Breakfast cereals	30	2	60	2416	145
Potatoes	200	2	482	287	138
**Food group 4—pulses**					
Dry pulses	50	3	150	4271	641
**Food group 5—fats**					
Vegetable oil	10	14	140	9631	1348
Butter	10	7	70	5553	389
**Food groups 6 and 7—fruit and vegetables**					
Fresh fruit	150	21	4074	819.2	3537
Nuts, shelled	30	2	60	10,244.6	615
Leafy vegetables	80	7	742	255.3	189
Other vegetables	200	10.5	2919	402	1173
**Indulgence food**					
Sugar	5	10.5	52.5	1351	71
Jam	20	2	40	2675	107
**Total**					**19,797**

^a^ Converted considering the percentage of the edible part, when needed.

**Table 3:** Water footprint of the high-energy (2500 kcal/day) dietary pattern.

Food Category	Serving Size (g of Edible Portion)	Suggested Consumption (Serving/Week)	Suggested Consumption (g/Week) ^a^	WFP (L/kg or L/L)	WF of the Suggested Consumption (L/Week)
**Food group 1—meat (terrestrial and aquatic animals) and eggs**					
Red meat	100	1	113	10,701	1209
White meat	100	3	429	4325	1855
Fish/seafood	150	3	642	2543	816
Processed fish	50	1	50	2543	64
Eggs	50	4	230	3265	751
**Food group 2—milk and dairy**					
Milk/yogurt	125	21	2625	1020	2678
Soft cheese	100	2	200	5060	1012
Hard cheese	50	1	50	5060	253
**Food group 3—cereals and potatoes**					
Bread	50	31.5	1575	1608	2533
Bread analogue	30	1	30	1608	48
Pasta, rice, barley	80	10.5	840	2108	1770
Sweet bakery (croissant)	50	1	50	4062	203
Sweet bakery (biscuits)	30	1	30	3057	92
Breakfast cereals	30	3	90	2416	217
Potatoes	200	2	482	287	138
**Food group 4—pulses**					
Dry pulses	50	3	150	4271	641
**Food group 5—fats**					
Vegetable oil	10	21	210	9631	2023
Butter	10	10	70	5553	389
**Food groups 6 and 7—fruit and vegetables**					
Fresh fruit	150	21	4074	819	3337
Nuts, shelled	30	2.5	75	10,244.6	768
Leafy vegetables	80	7	742	255.3	189
Other vegetables	200	14	3892	402	1565
**Indulgence food**					
Sugar	5	14	70	1351	95
Jam	20	2	40	2675	107
**Total**					**22,753**

^a^ Converted considering the percentage of the edible part, when needed.

## Data Availability

The archived data and all elaboration and analysis generated and used for the presentation of results in this study are fully available upon request from the corresponding author.

## References

[1] FAO (2012). Sustainable Diets and Biodiversity: Directions and Solutions for Policy, Research and Action.

[2] Heller M.C., Keoleian G.A., Willett W.C. (2013). Toward a life cycle-based, diet level framework for food environmental impact and nutritional quality assessment: A critical review. Environ. Sci. Technol..

[3] HLPE (2017). Nutrition and Food Systems. A Report by the High Level Panel of Experts on Food Security and Nutrition of the Committee on World Food Security.

[4] Chaudhary A., Gustafson D., Mathys A. (2018). Multi-indicator sustainability assessment of global food systems. Nat. Commun..

[5] Institute of Medicine (2000). Food and Nutrition Board. Dietary Reference Intakes.

[6] Società Italiana di Nutrizione Umana (2014). Livelli di Assunzione di Riferimento di Nutrienti ed Energia per la Popolazione Italiana (LARN) IV Revisione.

[7] Rossi L., Berni Canani S., Censi L., Gennaro L., Leclercq C., Scognamiglio U., Sette S., Ghiselli A. (2022). The 2018 Revision of Italian Dietary Guidelines: Development Process, Novelties, Main Recommendations, and Policy Implications. Front. Nutr..

[8] Rossi L., Ferrari M., Ghiselli A. (2023). The Alignment of Recommendations of Dietary Guidelines with Sustainability Aspects: Lessons Learned from Italy’s Example and Proposals for Future Development. Nutrients.

[9] James-Martin G., Baird D.L., Hendrie G.A., Bogard J., Anastasiou K., Brooker P.G., Wiggins B., Williams G., Herrero M., Lawrence M. (2022). Environmental Sustainability in National Food-Based Dietary Guidelines: A Global Review. Lancet Planet. Health.

[10] Vieux F., Soler L.G., Touazi D., Darmon N. (2013). High nutritional quality is not associated with low greenhouse gas emissions in self-selected diets of French adults. Am. J. Clin. Nutr..

[11] Jarmul S., Dangour A.D., Green R., Liew Z., Haines A., Scheelbeek P.F.D. (2020). Climate change mitigation through dietary change: A systematic review of empirical and modelling studies on the environmental footprints and health effects of ‘sustainable diets’. Environ. Res. Lett..

[12] Mekonnen M.M., Hoekstra A.Y. (2011). The green, blue and grey water footprint of crops and derived crop products. HESS.

[13] Mekonnen M., Hoekstra A.Y. (2012). A global assessment of the water footprint of farm animal products. Ecosystems.

[14] CREANUT Tabelle di Composizione Degli Alimenti, Aggiornamento 2019—Website Edited by L. Marletta & E. Camilli. https://www.alimentinutrizione.it/sezioni/tabelle-nutrizionali.

[15] Bostock J., McAndrew B., Richards R., Jauncey K., Telfer T., Lorenzen K., Little D., Lindsay R., Handisyde N., Gatward I. (2010). Aquaculture: Global status and trends. Phil. Trans. R. Soc. B.

[16] Yuan Q., Song G., Fullana-i-Palmer P., Wang Y., Semakula H.M., Mekonnen M.M., Zhang S. (2017). Water footprint of feed required by farmed fish in China based on a Monte Carlo-supported von Bertalanffy growth model: A policy implication. J. Clean. Prod..

[17] Harris F., Moss C., Joy E.J.M., Quinn R., Scheelbeek P.F.D., Dangour A.D., Green R. (2020). The Water Footprint of Diets: A Global Systematic Review and Meta-Analysis. Adv. Nutr..

[18] Vanham D., Guenther S., Ros-Baró M., Bach-Faig A. (2021). Which diet has the lower water footprint in Mediterranean countries?. Resour. Conserv. Recycl..

[19] Willett W., Rockström J., Loken B., Springmann M., Lang T., Vermeulen S. (2019). Food in the Anthropocene: The EAT-Lancet Commission on healthy diets from sustainable food systems. Lancet.

[20] Scholz-Ahrens K.E., Ahrens F., Barth C.A. (2020). Nutritional and health attributes of milk and milk imitations. Eur. J. Nutr..

[21] Thorning T.K., Raben A., Tholstrup T., Soedamah-Muthu S.S., Givens I., Astrup A. (2016). Milk and dairy products: Good or bad for human health? An assessment of the totality of scientific evidence. Food Nutr. Res..

[22] Hallström E., Carlsson-Kanyama A., Börjesson P. (2015). Environmental impact of dietary change: A systematic review. J. Clean. Prod..

[23] Aleksandrowicz L., Green R., Joy E.J., Smith P., Haines A. (2016). The impacts of dietary change on greenhouse gas emissions, land use, water use, and health: A systematic review. PLoS ONE.

[24] Ridoutt B.G., Hendrie G.A., Noakes M. (2017). Dietary strategies to reduce environmental impact: A critical review of the evidence base. Adv. Nutr..

[25] Vanham D., Leip A. (2020). Sustainable food system policies need to address environmental pressures and impacts: The example of water use and water stress. Sci. Total Environ..

[26] Bleischwitz R., Spataru C., VanDeveer S.D., Obersteiner M., van der Voet E., Johnson C., Andrews-Speed P., Boersma T., Hoff H., van Vuuren D.P. (2018). Resource nexus perspectives towards the United Nations Sustainable Development Goals. Nat. Sustain..

[27] Markantonis V., Reynaud A., Karabulut A., El Hajj R., Altinbilek D., Awad I.M., Bruggeman A., Constantianos V., Mysiak J., Lamaddalena N. (2019). Can the implementation of the water-energy-food nexus support economic growth in the Mediterranean region? The current status and the way forward. Front. Environ. Sci..

[28] Hachem F., Vanham D., Moreno L.A. (2020). Territorial and Sustainable Healthy Diets. Food Nutr. Bull..

[29] Mekonnen M.M., Hoekstra A.Y. (2016). Four billion people facing severe water scarcity. Sci. Adv..

[30] Bôto J.M., Rocha A., Miguéis V., Meireles M., Neto B. (2022). Sustainability Dimensions of the Mediterranean Diet: A Systematic Review of the Indicators Used and Its Results. Adv. Nutr..

[31] Springmann M., Clark M., Mason-D’Croz D., Wiebe K., Bodirsky B.L., Lassaletta L., de Vries W., Vermeulen S.J., Herrero M., Carlson K.M. (2018). Options for keeping the food system within environmental limits. Nature.

[32] Annunziata A., Mariani A. (2018). Consumer Perception of Sustainability Attributes in Organic and Local Food. Recent Pat. Food. Nutr. Agric..

[33] Jacobs S., Sioen I., Marques A., Verbeke W. (2018). Consumer response to health and environmental sustainability information regarding seafood consumption. Environ. Res..

[34] Schiano A.N., Harwood W.S., Gerard P.D., Drake M.A. (2020). Consumer perception of the sustainability of dairy products and plant-based dairy alternatives. J. Dairy Sci..

[35] Joyce A., Goddek S., Kotzen B., Wuertz S., Goddek S., Joyce A., Kotzen B., Burnell G.M. (2019). Aquaponics: Closing the Cycle on Limited Water, Land and Nutrient Resources. Aquaponics Food Production Systems.

